# Activity of MCPIP1 RNase in tumor associated processes

**DOI:** 10.1186/s13046-019-1430-6

**Published:** 2019-10-21

**Authors:** Katarzyna Miekus, Jerzy Kotlinowski, Agata Lichawska-Cieslar, Janusz Rys, Jolanta Jura

**Affiliations:** 1 Department of General Biochemistry, Faculty of Biochemistry, Biophysics, and Biotechnology, Jagiellonian University, Gronostajowa 7 Street, 30-387 Krakow, Poland; 2 Department of Tumour Pathology, Maria Skłodowska-Curie Memorial Center and Institute of Oncology, Krakow, Poland

**Keywords:** RNase, Regnase-1, Apoptosis, Proliferation, miRNA, Transcript stability

## Abstract

The monocyte chemoattractant protein-induced protein (MCPIP) family consists of 4 members (MCPIP1–4) encoded by the *ZC3h12A-D* genes, which are located at different loci. The common features of MCPIP proteins are the zinc finger domain, consisting of three cysteines and one histidine (CCCH), and the N-terminal domain of the PilT protein (PilT-N-terminal domain (PIN domain)). All family members act as endonucleases controlling the half-life of mRNA and microRNA (miRNA). The best-studied member of this family is MCPIP1 (also known as Regnase-1).

In this review, we discuss the current knowledge on the role of MCPIP1 in cancer-related processes. Because the characteristics of MCPIP1 as a fundamental negative regulator of immune processes have been comprehensively described in numerous studies, we focus on the function of MCPIP1 in modulating apoptosis, angiogenesis and metastasis.

## Background

The MCPIP family consists of four proteins (MCPIP1–4) encoded by four genes (*Zc3h12a*-*d* in mice and *ZC3H12A*-*D* in humans). MCPIP family members are multidomain proteins; however, two of the domains—the zinc finger domain and the PIN domain—determine their function. A total of 55 proteins that contain CCCH zinc finger domains are found in humans [1]. Most CCCH zinc finger proteins with known functions act as regulators of RNA metabolic processes, including mRNA splicing, polyadenylation, export, translation, and decay [2].

PIN domains are approximately 130 amino acids in length, and proteins possessing this domain function as nuclease enzymes that cleave single-stranded RNA (ssRNA) in a sequence-independent manner. The name “PIN domain” derives from the presence of such a domain at the N-terminus of an annotated type IV pili twitching motility (PilT) protein (the PilT N-terminal domain, or PIN domain). Proteins with PIN domains are present in all kingdoms of life and act in a metal-dependent manner, usually via Mg^2+^ or Mn^2+^ [3–6].

All MCPIP family members have been shown to possess an active PIN domain and to be involved in inflammatory processes, although MCPIP1 is the most well-studied and well-described family member. In this review, we focus entirely on the role played by MCPIP1 in tumour-associated processes. The central part of this review is intended to summarize our current understanding about the role of MCPIP1 in cancer development and progression. Recent advances in elucidating the molecular mechanism of MCPIP1 action have shed new light on its fundamental immunomodulatory function. Importantly, negative regulation of inflammatory reactions is already widely discussed; thus, in this review, we concentrate on cancer-related processes regulated by MCPIP1.

**Fig. 1:**
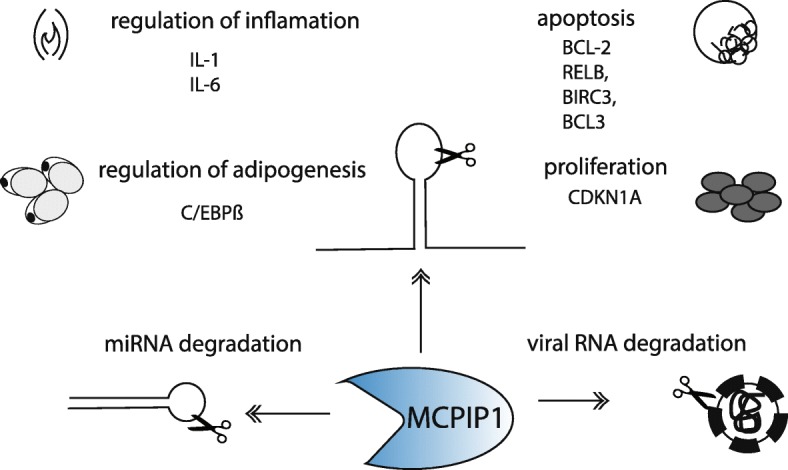
MCPIP1 regulates number of processes directly. MCPIP1 physically interacts with stem-loop structures in the 3′ UTR of transcripts and participates in their degradation. Destabilization of mRNA upon endonucleolytic cleavage by MCPIP1 leads to diminished protein translation and influences on inflammation, adipogenesis, proliferation and apoptosis. MCPIP1 degrades also miRNA by cleaving the terminal loops of precursor miRNAs and influences gene expression

MCPIP1 participates in the degradation of transcripts by recognizing specific stem-loop structures present in their 3′ untranslated regions (UTRs) (Fig. 1). Our recent studies showed that MCPIP1 recognizes a set of common target mRNAs encoding proteins that play important roles throughout the course of inflammation.

In addition to mediating direct endonucleolytic cleavage of RNA molecules, MCPIP1 is also involved in protein deubiquitination. By forming a complex with the TANK and USP10 proteins, MCPIP1 plays an indirect role in the deubiquitination of TRAF6. Via TANK-MCPIP1-USP10 complex activity, ubiquitin residues are removed from TRAF6 proteins by the USP10 deubiquitinase [7].

## Main text

### Mechanism of transcript degradation by MCPIP1

The level of mRNA in the cell results from competition between mRNA degradation and translation initiation. Mammalian cells contain two machineries by which RNA molecules are degraded: P-bodies (PBs) and stress granules (SGs). PBs and SGs can be clearly distinguished from each other by specific protein or RNA markers; however, they also share many proteins and mRNA species [8].

PBs are dynamic complexes whose assembly is dependent on the pool of nontranslated mRNA [9–11]. PBs contain a conserved core of proteins involved in mRNA decay and translational repression, such as the decapping enzyme complex, translational repressors and 5′ to 3′ exonucleases (reviewed in [12, 13]). SGs, also called mRNA silencing foci, were initially described in 1984 in tomato cell cultures as reversible aggregates of ribonucleoprotein complexes containing untranslated mRNA [14]. Later, similar structures were described in mammalian cells [15]. SGs are formed when global protein synthesis is inhibited in response to many different types of stress, such as UV irradiation, oxidative stress, and energy depletion. SGs are tightly associated with components of the translation machinery.

There are three major classes of intracellular RNA-degrading enzymes (ribonucleases or RNases): endonucleases, which cut RNA internally; 5′ exonucleases, which hydrolyse RNA from the 5′ end; and 3′ exonucleases, which degrade RNA from the 3′ end. Most RNases exhibit overlapping activities that result in redundancy of RNA degradation systems. Thus, multiple enzymes can recognize the same target RNAs (reviewed in [16]). RNases recognize *cis*-regulatory elements (CREs) in mRNA, such as secondary structures [17, 18] or sequence motifs present in the 3′ UTR including binding sites of RNA-binding proteins (RBPs) [19, 20].

MCPIP1 degrades transcripts in an adenine-uridine element (ARE)-independent manner [21–23]. MCPIP1 physically interacts with stem-loop structures in the 3′ UTR of transcripts through its PIN domain, causing mRNA destabilization followed by degradation [24]. High-throughput sequencing of RNA isolated by crosslinking immunoprecipitation (HITS-CLIP) revealed that the stem-loop sequences preferably recognized by MCPIP1 contain pyrimidine-purine-pyrimidine (YRY) loop motifs [24]. However, many transcripts identified experimentally, both by our group and by other research teams, do not contain a YRY motif. RNA immunoprecipitation assays and functional assays on an MCPIP1 mutant with a mutated PIN domain showed that these transcripts interact with MCPIP1 and that their levels depend on the active form of MCPIP1 (Fig. 1) [25].

*In vitro* analysis of recombinant MCPIP1 and oligonucleotides forming stem-loops from the 3′ UTR of IL-6 mRNA showed that cleavage occurs at the loop site of the stem-loop. Thus, the stem-loop structure is destabilized, and ssRNA fragments are generated, which are further progressively degraded through the following steps. MCPIP1 cleaves diverse sets of RNA stem-loop structures without a specific sequence preference [25]. However, the mechanism by which MCPIP1 recognizes its substrates in vivo remains to be clarified. Interactors and/or posttranslational modifications of MCPIP1 may affect its substrate specificity. In addition, size exclusion chromatography of the MCPIP1 and PIN proteins revealed that MCPIP1 undergoes homooligomerization during interaction with RNA substrates [25].

Importantly, MCPIP1 not only downregulates a set of mRNAs but also acts as a suppressor of miRNA biogenesis by cleaving the terminal loops of precursor miRNAs, thus counteracting Dicer1 activity [26]. Although MCPIP1 degrades miRNA and mRNA through the same mechanism by recognizing specific structures in both types of RNA targets, whether this protein is present in PBs, SGs or both structures depending on circumstances is unclear.

### The role of MCPIP1 in apoptosis

The role of MCPIP1 in the regulation of cell death was originally demonstrated in human embryonic kidney (HEK) 293 cells and cardiomyocytes (Tables 1 and 2). Despite accumulating evidence supporting the proapoptotic role of MCPIP1, knowledge of the means by which it induces cell death is still very limited. Moreover, MCPIP1 may regulate the apoptotic process both directly and indirectly [39]. The indirect effect of MCPIP1 on apoptosis is connected to its influence on the formation of SGs [40]. Expression of MCPIP1 completely blocked SG formation and promoted macrophage apoptosis under stress conditions, including arsenite-induced oxidative stress, heat shock, and energy deprivation [40]. Consistent with these findings, MCPIP1-deficient cells (splenocytes and murine embryonic fibroblasts) spontaneously formed SG aggregates even in the absence of stress and displayed apoptosis resistance. In addition, elevated levels of MCPIP1 were detected in ischaemic human hearts—in situ hybridization showed the presence of MCPIP1 transcripts, and immunohistochemistry demonstrated that the MCPIP1 protein colocalized with apoptotic nuclei [39].

**Table 1:** Effect of MCPIP1 on gene expression. Regulation of genes expression and proteins level by MCPIP1 was tested both in cells with *ZC3H12A* overexpression or silencing. Cited results were obtained from studies using cells cultured in control conditions. We did not include data obtained upon induction of differentiation (i.e. adipogenesis), nor stimulation (i.e. cytokines, LPS)

MCPIP1 OVEREXPRESSION		
gene expression	Experimental model	Reference
HIF-1α, VEGF1, cdh12, cdh19, VE-cadherin^a^	HUVECs	Niu et al., 2008 [27]
Ephrin-A1, IL-1β, Notch Homolog 4, Ephrin B2, PDGF α, TIMP-2, Ephrin A3, Midkine, Thrombospondin 1, CSF-3^b^		
Flt-1, Flk-1, Tie-2, CD31, Beclin-1^a^	bone marrow mononuclear cells	Niu et al., 2013 [28]
VEGF, COX2, SIRT-1^a^	HUVECs	Roy et al., 2013 [29]
Fas, Dedd2^a^	MDA-MB-231	Lu et al., 2016 [30]
MBLN2, SLC3A2, DFFB, APAF1^a^	BE(2)-C human neuroblastoma cell line	Boratyn et al., 2016 [31]
CDKN1A^a, c^	ccRCC cell line Caki-1	Lichawska-Cieslar et al., 2018 [32]
gene expression	Experimental model	Reference
CD14, CD11b^a^	bone marrow mononuclear cells	Niu et al., 2013 [28]
TSP-1 and VEGI, p65	HUVECs	Roy et al., 2013 [29]
Bcl2L1, Bcl2A1, Birc3, RelB, and Bcl3^a^	MDA-MB-231	Lu et al., 2016 [30]
CTXN1, CNIH2, MCM10, CD248, RBM12, PAXIP1, SEPT3^a^	BE(2)-C human neuroblastoma cell line	Boratyn et al., 2016 [31]
SLC44A1, SLC29A4,		
IL-6, VEGF, GLUT-1^a^	ccRCC cell line Caki-1	Ligeza et al., 2017 [33]
c/EBPβ, SDF-1, Snail, Zeb2^a^	ccRCC cell line Caki-1	Marona et al., 2017 [34]
HSPA5, AGR2, PLOD2, MMP2, NDRG1, NDRG2, SPHK1, ENPP2,	ccRCC cell line Caki-1	Lichawska-Cieslar et al., 2018 [32]
NGEF, GPRC5B, TSC22D3, SGK2, FRAT1, RIPK4, DDB1 ^a, c^		
MCPIP1 SILENCING		
gene expression	Experimental model	Reference
BCL2L1, BCL3, BIRC3, RELB, AND BCL2A1	MDA-MB-231	Lu et al., 2016 [30]
IL-8, VEGF, IL-6, c/EBPβ, SDF-1, CXCR4, Snail, Zeb2^a^	ccRCC cell line Caki-1	Marona et al., 2017 [34]

^a^Real-time PCR analysis

^b^Gene array analysis; expression profile of angiogenesis-related genes in GFP/hMCPIP-over GFP-infected HUVECs with fold induction > 5

^c^RNA-Seq analysis

**Table 2:** Effect of MCPIP1 on gene expression. Regulation of genes expression and proteins level by MCPIP1 was tested both in cells with *ZC3H12A* overexpression or silencing. Cited results were obtained from studies using cells cultured in control conditions. We did not include data obtained upon induction of differentiation (i.e. adipogenesis), nor stimulation (i.e. cytokines, LPS)

MCPIP1 OVEREXPRESSION		
protein level	Experimental model	Reference
cdh12, cdh19, VE-cadherin^a^	HUVECs	Niu et al., 2008 [27]
Flt-1, Flk-1, Tie-2, VEGF, Beclin-1^a^	bone marrow mononuclear cells	Niu et al., 2013 [28]
CD31, VE-cadherin^b^		
HIF1α, SIRT-1^a^	HUVECs	Roy, 2013 [29]
Ras-related protein Rab-11B, Testin^c, e^	mesenchymal stem cells	Labedz-Maslowska et al., 2015 [35]
Endothelin, IP-10, TIPM-1, MMP-3, NOV^a^		
Pro-Caspase-3^a^	MDA-MB-231, 4 T1 cell line	Lu et al., 2016 [30]
PARP1^a^	4 T1	Lu et al., 2016 [30]
DFFB, APAF1^a^	BE(2)-C human neuroblastoma cell line	Boratyn et al., 2016 [31]
E-cadherin^a^	ccRCC cell line Caki-1	Marona et al., 2017 [34]
p21^a^	ccRCC cell line Caki-1	Lichawska-Cieslar et al., 2018 [32]
protein level	Experimental model	Reference
PAI-1, iNOS^a^	HUVECs	Qi et al., 2010 [36]
TSP-1, VEGI, p65^a^	HUVECs	Roy et al., 2013 [29]
CAAX prenyl protease 1 homolog, Calumenin^c, f^ KC^a^	mesenchymal stem cells	Labedz-Maslowska et al., 2015 [35]
HIF1α, HIF2α^a^	ccRCC cell line Caki-1	Ligeza et al., 2017 [33]
c-Met, Src, βcatenin, Vimentin, c/EBPβ^a^	ccRCC cell line Caki-1	Marona et al., 2017 [34]
p53, p21^a^	human primary keratinocytes	Bugara et al., 2017 [37]
MCPIP1 SILENCING		
protein level	Experimental model	Reference
c-Met, Src, βcatenin, Vimentin^a^ c/EBPβ^a^	ccRCC cell line Caki-1	Marona et al., 2017 [34]
IL-8, VEGF, IL-6^d^		
Cyclin D1^a^	human primary keratinocytes	Bugara et al., 2017 [37]
IL-8^d^		
VCAM-1^a^	HUVECs	Li et al., 2018b [38]
protein level	Experimental model	reference
E-cadherin^a^	ccRCC cell line Caki-1	Marona et al., 2017 [34]

^a^western blot analysis

^b^immunofluorescence analysis

^c^proteomic analysis by mass spectroscopy

^d^ELISA

^e^fold change > 5

^f^fold change <−5

These findings are consistent with experiments performed in Caki-1 cells as a model of clear cell renal cell carcinoma (ccRCC). Overexpression of MCPIP1 reduced cell viability, induced nuclear morphology characteristic of late apoptosis and enhanced caspase 3/7 activity [33].

The proapoptotic properties of MCPIP1 are also triggered by its involvement in pre-miRNA degradation (Table 3). Boratyn and coworkers showed that overexpression of MCPIP1 in the BE(2)-C human neuroblastoma cell line resulted in a significant reduction in miR-3613-3p levels [31]. Further investigation indicated that in those cells, miRNA-3613-3p overexpression negatively regulated the expression of apoptotic protease activating factor 1 (APAF1) [41]. Overexpression of wild-type but not mutated MCPIP1 (with deletion of the PIN domain) in BE(2)-C cells resulted in miR-3613-3p downregulation and significant increases in pro-apoptotic DFFB and APAF1 at the mRNA and protein levels [31]. Thus, in several cancer cells characterized by low levels of MCPIP1, upregulated miR-3613-3p may decrease the possibility of apoptosis activation, whereas BE(2)-C cells overexpressing miR-3613-3p exhibit inhibition of caspase-9 proteolysis [41].

**Table 3:** Effect of MCPIP1 on miRNA expression. Selection of positively and negatively regulated miRNA by MCPIP1. Negative regulation of miRNA by MCPIP1 was analyzed either by overexpression of ZC3H12A (more MCPIP1 protein leads to diminished amount of miRNA), or ZC3H12A silencing (less MCPIP1 protein results in miRNA accumulation)

MCPIP1		
miRNA	Experimental model	Reference
miR-155^a^	Jurkat T cells	Suzuki et al., 2011 [26]
miR-16^a^	THP-1	Suzuki et al., 2011 [26]
miR-21, −26a, −107, − 182, −146a, −17-5p, −135b, let-7 g^a^	HepG2, HEK293T	Suzuki et al., 2011 [26]
miR-20b, miR-34a^b^	HUVECs	Roy et al., 2013 [29]
miR-3613-3p^b^	BE(2)-C human neuroblastoma cell line	Boratyn et al., 2016 [31]

^a^silencing of MCPIP1 increases miRNA level

^b^overexpression of MCPIP1 decreases miRNA level

On the other hand, a study by Oh and coworkers showed an antiapoptotic role of MCPIP1 mediated via regulation of apoptosis-related death receptor 5 (DR5). DR5 is a cell surface receptor produced endogenously by various immune cells, such as T cells and is responsible for TNF-related apoptosis. MCPIP1 decreases both the total cellular and cell surface expression of DR5, primarily through modulating DR5 autophagic/lysosomal degradation. Mechanistically, the authors implicated indirect MCPIP1 action, showing the involvement of this protein in deubiquitination, which leads to decreased DR5 stability. In addition, suppression of MCPIP1 by gene knockdown enhanced TRAIL- or DR5-induced apoptosis in cancer cells, as manifested by the activation of caspase 3 and 8 and subsequent DNA fragmentation [42].

MCPIP1 also regulates apoptosis directly via a mechanism directly linked to its enzymatic activity (Tables 1 and 2) [30]. Studies in the breast cancer cell line MDA-MB-231 indicated that MCPIP1 functions as a potent tumour suppressor that induces apoptosis by selectively enhancing the decay of antiapoptotic gene mRNA transcripts. Lu and coworkers identified 31 transcripts affected by MCPIP1 expression, of which 6 antiapoptotic genes were downregulated and 25 proapoptotic genes were upregulated [30]. RNA immunoprecipitation experiments demonstrated that MCPIP1 directly binds and cleaves mRNAs encoding Bcl2L1, Bcl2A1, RelB, Birc3, and Bcl3. Finally, analysis of human samples revealed that MCPIP1 expression is suppressed in breast tumour cells, which, in turn, may help these cells evade apoptosis [30].

### The antiproliferative function of MCPIP1

Similar to resistance to cell death, sustained proliferative signalling is another important hallmark of cancer. MCPIP1 is primarily known as a negative regulator of inflammation; however, it also regulates cell proliferation. The first interesting observation came from a study performed by Lu and coworkers, who demonstrated decreased MCPIP1 protein and RNA levels in breast cancer specimens [30]. Additionally, MCPIP1 inhibited the proliferation of breast cancer cells both in vitro and in vivo. The authors proved that MCPIP1 suppressed the growth of breast tumours in vivo by inhibiting cell proliferation and concomitantly inducing apoptosis. Inoculation of MDA-MB-231/Tet-On tumour cells into the mammary glands of immunocompromised mice allowed the study of tumour growth upon MCPIP1 overexpression. The day after MCPIP1 induction with doxycycline in the tumour-bearing mice, the tumours started to shrink and then rapidly disappeared within 6 days, but the tumours in control mice continued to grow [30].

A low level of MCPIP1 is also a signature of ccRCC [33]. MCPIP1 expression varies depending on the tumour grade and decreases significantly with tumour progression, which suggests that MCPIP1 is involved in cancer growth and metastasis [34]. Studies performed in the ccRCC cell lines Caki-1 (metastatic) and Caki-2 (primary tumour) strongly support the antiproliferative function of MCPIP1 [32, 34]. MCPIP1 depletion in ccRCC cells significantly enhanced tumour cell proliferation in both examined cell lines, Caki-1 and Caki-2.

The antiproliferative action of MCPIP1 was also confirmed in animal studies. The growth of human ccRCC was assessed in an in vivo xenotransplantation model established in NOD-SCID mice via subcutaneous injection of Caki-1 cells. These experiments proved that inhibition of MCPIP1 in Caki-1 cells affected both tumour growth and weight. The effect was opposite when cells with MCPIP1 overexpression were used [34].

Moreover, data from human neuroblastoma biopsies were even more unambiguous, since MCPIP1 transcription was not detected in any sample from the 29 specimens analysed by Skalniak and coworkers [43]. Similar to primary tumours, human neuroblastoma cell lines exhibited low protein levels of MCPIP1, and overexpression of the *ZC3H12A* gene in BE(2)-C cells caused a significant decrease in cell viability and proliferation [43].

One mechanism explaining the influence of MCPIP1 on the proliferation rate is the involvement of this RNase with p21^Cip1^ (CDKN1A) mRNA. Caki-1 cells expressing MCPIP1 showed significantly higher expression of p21^Cip1^ protein and mRNA than control and D141N cells (with a point mutation in MCPIP1 resulting in an inactive catalytic site). The p21^Cip1^ protein belongs to the Cip/Kip family of inhibitors and blocks the cell cycle by inhibiting Cyclin-Cdk complexes. During S phase, p21^Cip1^ degradation is regulated by the activity of the Cul4-DDB1-Cdt2 E3 ligase. In our RNA-Seq analysis, the transcript levels of damage-specific DNA binding protein 1 (DDB1) were reduced in MCPIP1-expressing cells compared with control cells or cells expressing MCPIP1 with an inactive PIN domain (D141N). The RNase activity of MCPIP1 is indispensable for the degradation of DDB1 transcripts, which in turn may lead to p21^Cip1^ accumulation. Thus, MCPIP1 inhibits the cell cycle progression and growth of Caki-1 cells by upregulating the cell cycle inhibitor p21^Cip1^ [32]. In addition, siRNA silencing of MCPIP1 in human primary keratinocytes was shown to decrease the levels of phosphorylated p53 and p21 proteins and to upregulate Cyclin D1 expression after exposure to UVB radiation stress, which may serve as a mechanism of survival promotion in MCPIP1-depleted cells [37].

Additionally, MCPIP1 controls the proliferation rate and tumorigenesis by controlling the half-life of miR-155 (Table 3) [26]. MCPIP1 was initially described to modulate the immune response via the miR-155/c-Maf axis [26]. In addition to acting as a regulator of the immune response, miR-155 is thoroughly described as an oncogenic miRNA (oncomiR) that contributes to the development of leukaemia and breast, lung and stomach tumours. MiR-155 was described to promote tumorigenesis by targeting several factors, thus enhancing proliferation, granting resistance to cell death (reviewed in [44]) and inducing angiogenesis [45]. As already discussed, the expression of MCPIP1 was reported to be downregulated in several carcinoma types, including breast cancer, neuroblastoma, and ccRCC. Upregulation of miR-155 is a potential MCPIP1-dependent effect contributing to the promotion of tumorigenesis. The regulation of another cancer-related miRNA, miR-146a, by MCPIP1 was investigated by several groups [26, 46, 47]. A study by Qu and coauthors showed that MCPIP1 weakens the LPS induction of miR-146a in THP-1 cells treated with type I interferon (IFN) [46]. The miR-146a targets include several factors crucial for proinflammatory signalling (e.g., tumour necrosis factor receptor-associated factor 6 (TRAF6) and interleukin-1 receptor-associated kinase (IRAK-1)) [48], and hence, miR-146a deficiency in the white blood cells of systemic lupus erythematosus patients is correlated with upregulation of MCPIP1 expression and overactivation of inflammatory responses [46]. Thus, MCPIP1 regulates the expression of both miR-155 and miR-146a, which are important modulators of immune processes and tumorigenesis. However, those miRNAs usually exert opposing roles in the regulation of the immune functions, and their expression is often deregulated in tumours [48, 49].

### The role of MCPIP1 in the regulation of angiogenesis

The formation of tumour-associated vasculature (i.e., tumour angiogenesis) has emerged as a critical step promoting local tumour progression and metastatic spread. Accumulating evidence indicates that MCPIP1 plays a role during the process of angiogenesis in regulating inflammation, transcription factor activity, the production of angiogenic factors and miRNA biosynthesis. However, studies in tumour cells indicate that MCPIP1 may exhibit diverse actions under normal and pathological conditions.

Inflammation is a major inducer of angiogenesis during tumour progression [50], and inflammatory cytokines have been reported to facilitate a broad spectrum of tumour development processes. The proinflammatory cytokines IL-1, IL-6 and monocyte chemotactic protein-1 (MCP-1) are required for angiogenesis and tumour growth and promote the invasion and metastasis of cancer cells in animal models. The first studies of the role of MCPIP1 in the process of angiogenesis showed that the treatment of human umbilical vein endothelial cells (HUVECs) with the inflammatory agents TNF-α, IL-1β, IL-8 and MCP-1 increased expression of gene coding for MCPIP1, which subsequently induced angiogenesis-related properties and the expression of angiogenesis-related genes, resulting in capillary-like tube formation (Tables 1 and 2) [27, 51]. Moreover, forced MCPIP1 expression causes oxidative and nitrosative stress, resulting in ER stress and ultimately leading to autophagy, which is required for angiogenesis [51].

In addition, the influence of MCPIP1 on the acquisition of angiogenic properties has been documented in different types of cells (Tables 1 and 2). The Kollatukudy group showed that MCPIP1 expression increased during MCP-1-induced transdifferentiation in human bone marrow mononuclear cells (BMNCs) [28]. MCPIP1 induced the acquisition of an endothelial cell-like morphology, downregulation of the monocytic markers CD14 and CD11b, upregulation of the endothelial markers Flk-1 and Tie-2, induction of cdh-12 and -19 expression, activation of ER stress, and autophagy [28]. These results demonstrate that MCPIP1 may be an important regulator of inflammatory angiogenesis.

Angiogenesis regulation is also tightly connected with the expression of adhesion molecules on the endothelial surface. Overexpression of MCPIP1 has been described to suppress VCAM-1 expression and monocyte adhesion to human endothelial cells. Conversely, knockdown of MCPIP1 increases cytokine-induced VCAM-1 expression in HUVECs and enhances monocyte adhesion [36]. Moreover, studies by the Fu group showed that increased MCPIP1 protein levels in endothelial cells resulting from the inhibition of MALT1 protease activity suppress endothelial activation. Moreover, correlations have been found between increased levels of MCPIP1 and both inhibition of TNFα-induced VCAM-1 expression in HUVECs and LPS-induced VCAM-1 expression in mice. In addition, inhibition of MALT1 protease activity significantly inhibits TNFα-induced adhesion of THP-1 monocytic cells to HUVECs [38].

MCPIP1 may play a key role in the vascularization process by controlling the levels of proangiogenic transcripts and proteins. Enhanced expression of MCPIP1 has been shown to increase the angiogenic capacity and the expression of proangiogenic genes, such as those encoding the intranuclear transcription factor Gata-2 and membrane VE-cadherin. These two genes allow mesenchymal stem cells (MSCs) to differentiate into endothelial cells. Additionally, MCPIP1-overexpressing MSCs secrete increased levels of endothelin, TIMP-1, Serpin E1, IFN-γ-inducible protein-10 (IP-10), MMP-3, stromal cell-derived factor 1 (SDF-1), osteopontin and insulin-like growth factor-binding protein 9 (Tables 1 and 2) [35]. Moreover, the Kollatukudy group [27] showed that transfection of HUVECs with an MCPIP-GFP expression vector induced HIF-1α and VEGF production, whereas silencing of MCPIP1 by siRNA suppressed MCP-1-induced expression of HIF-1α and VEGF. Angiogenesis gene array analysis revealed that MCPIP induced the upregulation of 31 of 113 genes known to contribute to the augmented angiogenic properties of endothelial cells (Tables 1 and 2) [27]. Further studies showed that low levels of MCPIP1 in ccRCC induce endothelial cell angiogenesis and that the lack of MCPIP1 RNase activity is responsible for the secretion of proangiogenic factors—VEGF, IL-8 and IL-6—by tumour cells [34].

The effect of MCPIP1 on vascularization might be triggered by the regulation of transcription factors such as HIFs or NF-κB. Indeed, Caki-1 cells overexpressing MCPIP1 exhibit diminished levels of HIF1α and HIF2α under hypoxic conditions [33]. The mechanism underlying the diminished level of HIF2α is based on a decrease in the half-life of the transcript coding for this protein. Consequently, cells overexpressing MCPIP1 display decreased expression levels of transcripts coding for VEGFA and IL-6 [33]. Inhibition of NF-κB activity by MCPIP1 leads to a reduction in the levels of NF-κB target genes, including those encoding antiangiogenic factors such as thrombospondin-1 (TSP-1) and VEGI, which are natural inhibitors of angiogenesis [29].

A study by Roy and coworkers indicated that the anti-Dicer RNase activity of MCPIP1 is also critical for modulating angiogenesis. In HUVECs, the expression of antiangiogenic miR-20b and miR-34a is MCPIP1-dependent [29]. Overexpression of wild-type MCPIP1 but not the RNase-dead mutant decreased the levels of miR-20b and miR-34a. Conversely, silencing of MCPIP1 upregulated miR-20b and miR-34a expression upon stimulation with TNF-α or IL1-β. These miRNAs affect the expression of HIF-1α and SIRT-1, which are the critical positive regulators of blood vessel formation. Specifically, miR-20b represses HIF-1α and miR-34a affects SIRT-1 translation. Roy and coworkers further showed that in HUVECs, overexpression of MCPIP1 induces tube formation, as described previously [27, 29]. This effect is, however, inhibited when MCPIP1 is cotransfected with either miR-20b or miR-34a mimetics, most likely via downregulation of HIF-1α and SIRT1 [29].

In addition to directly regulating proangiogenic cytokines, MCPIP1 can regulate angiogenesis indirectly. Overexpression of VEGF has been reported to stimulate angiogenesis by upregulating SDF-1, a chemotactic chemokine, thereby recruiting CXCR4-positive proangiogenic myeloid cells [52] and endothelial progenitor cells from the bone marrow [53]. In addition, SDF-1 and VEGF synergistically induce neoangiogenesis in tumours [54]. Studies in Caki-1 cells showed that MCPIP1 silencing increases SDF-1 expression both in vitro and in vivo and that the RNase activity of MCPIP1 controls the level of SDF-1 mRNA [34]*.* The CXCR4/SDF-1 axis can coordinate the metastasis of various tumours, and our observations not only demonstrate the impact of MCPIP1 on tumour angiogenesis but also highlight the role of MCPIP1 downregulation in potentiating SDF-1-CXCR4 signalling.

**Fig. 2:**
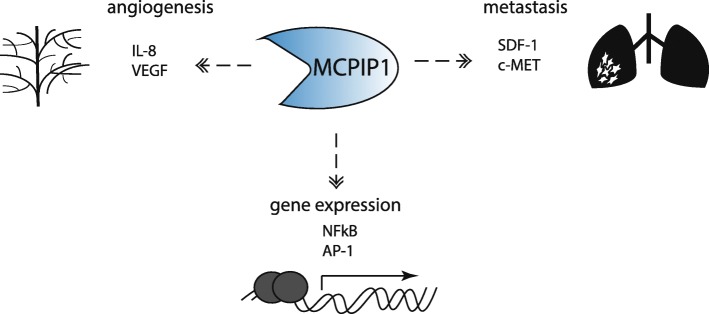
Mechanisms of indirect MCPIP1 action. MCPIP1 plays important role in affecting angiogenesis or metastasis and transcription factors activity

The current research shows that MCPIP1 can regulate angiogenesis by different means in normal and tumour cells. In normal endothelial cells, MCPIP1 induces proangiogenic properties by stimulating the secretion of chemokines and growth factors. On the other hand, tumours are characterized by a low level of MCPIP1 and a well-developed tumour vasculature. This low level of MCPIP1 in tumour cells is correlated with a high level of proangiogenic factors, which activate endothelial cells to form blood vessels in progressing tumours (Fig. 2).

### MCPIP1 regulates tumour metastasis

One of the most dangerous features of malignant tumours, which are the most common cause of death in patients with diagnosed cancer, is the ability of tumour cells to metastasize. The critical stage in the process of metastasis is epithelial-to-mesenchymal transition (EMT), in which epithelial cells acquire mesenchymal features that facilitate their migration, invasion of neighbouring tissues and metastasis. During EMT, polarized epithelial cells, which have high expression levels of E-cadherin and other proteins characteristic of the epithelial cell phenotype, are influenced by growth factors, cytokines, and other environmental factors to undergo a change to an elongated morphology and become migrating cells expressing proteins such as vimentin, fibronectin, and N-cadherin, with a concomitant decrease in E-cadherin expression [55].

An important role of MCPIP1 in mediating the metastatic potential of cancer cell lines was shown in ccRCC cell lines. The decrease in MCPIP1 expression was correlated with the presence of the mesenchymal phenotype, which is essential for the metastatic process, and with a decrease in the E-cadherin level, an increase in the vimentin and β-catenin levels and a consequent increase in migration activity [34]. The reduction in the E-cadherin level was inversely correlated with the expression of the Snail and ZEB-2 transcription factors, which suppress E-cadherin expression [56, 57]. Moreover, silencing of MCPIP1 in ccRCC cells was associated with both an increased number of circulating tumour cells in mouse blood and augmented lung metastasis [34]*.*

Similar results were obtained by Lu and colleagues, who showed that the induction of MCPIP1 expression in MDA-MB-231/Tet-On tumour cells inoculated into the mammary glands of immunocompromised NSG mice significantly reduced tumour growth and lung metastasis. Moreover, MCPIP1 expression is inversely correlated with survival in breast cancer patients [30].

Moreover, MCPIP1 affects the expression level and phosphorylation of the c-Met (a mesenchymal-epithelial transition factor) receptor (Fig. 2). c-Met is a receptor tyrosine kinase that is expressed on the surface of various epithelial cells. The gene coding for c-Met is considered a protooncogene because abnormal activation of c-Met can promote the development and progression of multiple cancers, such as liver, lung, colon, breast, pancreatic, ovarian, prostate, and gastric carcinomas, in addition to cancers of the nervous system, such as glioblastoma [58–60]. MCPIP1 overexpression has been shown to reduce the expression and endogenous phosphorylation levels of c-Met and decrease the level of Src kinase in ccRCC [34]*.* The gene coding for C-Met is a direct target of NFκB, and MET participates in NFκB-mediated cell survival [61]. The regulation of NF-κB transcription factor activity by MCPIP1 could thus be expected to influence the level and function of the c-Met receptor but the regulation of the c-Met mRNA level by MCPIP1 needs to be clarified.

In a recent study of cell migration at the single-cell level, Zhuang and coauthors found that the expression of MCPIP1 is related to the mobility of cancer cells [62]. In particular, an inverse correlation between the migratory potential of the MCF-7, MDA-MB-231 and SUM-159 breast cell lines and the mRNA/protein expression of MCPIP1 was found. Transient transfection of MDA-MB-231 cells with a vector encoding MCPIP1 reduced cell mobility, and RNA-Seq of those cells revealed enrichment of TGF-β-suppressed genes in MCPIP1-overexpressing cells. The authors further showed that inhibiting TGF-β in MDA-MB-231 cells with low levels of MCPIP1 expression restored their migratory phenotype to that observed in the corresponding cells with high levels of MCPIP1 expression. This mechanism was further validated in an in vivo xenograft model, in which high MCPIP1 expression inhibited tumour growth and inhibit breast cancer invasion, while additional treatment of xenografts with low levels of MCPIP1 expression with a TGF-β inhibitor attenuated their growth phenotype. These results indicated that the inhibitory effect on cell migration and metastasis of MCPIP1 might be associated with the suppression of TGF-β signaling pathway [62].

MCPIP1 also controls the EMT process by negatively regulating the maturation of miRNA-200 family members, as shown in pancreatic adenocarcinoma [63]. In several pancreatic tumour cell lines, the MCPIP1/Dicer1 ratio and the levels of miRNA-200 family members are inversely correlated (Table 3). MiR-200 family members perform tumour suppressor functions, and their expression is frequently suppressed in cancer cells. These miRNAs regulate EMT by targeting ZEB1 and ZEB2, transcriptional repressors of E-cadherin. Decreased expression of miRNA-200 family members leads to upregulation of ZEB1/ZEB2 expression, promoting the mesenchymal-like state. This observation contrasts with the previously thoroughly described role of MCPIP1 in EMT regulation in renal carcinoma cells. However, the role of MCPIP1 in pancreatic adenocarcinomas has not yet been investigated.

Considering the current knowledge, MCPIP1-mediated control of the levels of transcription factors (NFκB and C/EBPβ) and signalling proteins (JNK and Akt) may play a key role in the activation and regulation of the EMT process [34, 62, 64].

## Conclusions

The interactions between *cis*-acting elements within mRNAs and trans-acting factors (RBPs) play a pivotal role in the posttranscriptional control of gene expression [65, 66]. RNA molecules are degraded by exo- and endonucleases that recognize specific sequences or structures in their targets. The MCPIP family of proteins are endonucleases that degrade mRNA transcripts by recognizing the stem-loop structure(s) at the 3′ UTR end of mRNA. Among MCPIPs, MCPIP1 is the best-described protein, and previous studies have shown that this protein regulates transcripts involved in processes such as inflammation, cell metabolism, angiogenesis, differentiation, proliferation and apoptosis. MCPIP1 degrades pri-miRNAs in addition to mRNAs. MCPIP1 activity results in control of the intracellular level of RNA and miRNA molecules. The exact list of transcripts is unknown and requires detailed study based on analysis of the interaction of MCPIP1 proteins with the RNA pool and sequencing of matrices purified after immunoprecipitation with antibodies specific for MCPIP1. MCPIP1 can regulate the level of transcripts directly by degrading them or indirectly by degrading the regulators of their expression, e.g., the mRNAs of transcription factors that regulate the expression of these transcripts, or by degrading specific miRNAs.

Further research is necessary to explain the factors that control the recognition of specific templates by MCPIP family members and determine the activation of a specific MCPIP under distinct physiological and pathological conditions. Furthermore, the correlations between MCPIP1 expression and tumour types and cancer stages need further investigation.

## Data Availability

Not applicable.

## Abbreviations

APAF1: Apoptotic protease activating factor 1
ARE: Adenine-uridine elementselement
BMNCs: Bone marrow mononuclear cells
C/EBP: CCAAT-enhancer-binding protein
CCCH: Three cysteines and one histidine
ccRCC: Clear cell renal cell carcinoma
CDKN1A: Cyclin Dependent Kinase Inhibitor 1A
CRE: Cis-regulatory elements
DDB1: Damage -specific DNA binding protein 1
DFFB: DNA fragmentation factor subunit β
DR5: Apoptosis-related death receptor 5
EMT: Epithelial- to- mesenchymal transition
HEK: Human embryonic kidney cells
HIF-1α: Hypoxia-inducible factor 1-alpha
HITS-CLIP: High-throughput sequencing of RNA isolated by crosslinking immunoprecipitation
HUVECs: Human umbilical vein endothelial cells
IFN: Interferon
IL-1: Interleukin 1
IL-6: Interleukin 6
IL-8: Interleukin 8
IRAK-1: Interleukin-1 receptor-associated kinase
LPS: Lipopolysaccharide
MCP-1: Monocyte chemotactic protein-1
MCPIP: Monocyte chemoattractant protein-induced protein
miRNAs: MicroRNAs
MMP3: Matrix Metalloprotease 3
mRNA: messenger RNA
MSCs: Mesenchymal stem cells
NFκB: Nuclear Factor kappa-light-chain-enhancer of activated B cells
NGS: Next-generation sequencing
NSG mouse: NOD scid gamma mouse
oncomiR: oncogenic miRNA
PBs: P-bodies
PIN domain: N-terminal domain of the PilT protein (PilT-N-terminal domain)
RBP: RNA-binding protein
SDF-1: Stromal cell-derived factor 1
SGs: Stress granules
siRNA: small interfering RNA
ssRNA: single-stranded RNA
TGF-β: Transforming growth factor β
TNF: Tumour necrosis factor
TRAF6: Tumour necrosis factor receptor-associated factor 6
UTR: 3′ untranslated region
VCAM-1: Vascular cell adhesion molecule 1
VEGF: Vascular endothelial growth factor
YRY: Pyrimidine-purine-pyrimidine
